# The Development of p53-Targeted Therapies for Human Cancers

**DOI:** 10.3390/cancers15143560

**Published:** 2023-07-10

**Authors:** Yier Lu, Meng Wu, Yang Xu, Lili Yu

**Affiliations:** 1 Department of Medical Oncology, Key Laboratory of Cancer Prevention and Intervention, Ministry of Education, The Second Affiliated Hospital, School of Medicine, Zhejiang University, Hangzhou 310009, China; 22118554@zju.edu.cn (Y.L.); 12118327@zju.edu.cn (M.W.); 2 Department of Cardiology, The Second Affiliated Hospital, Cardiovascular Key Lab of Zhejiang Province, School of Medicine, Zhejiang University, Hangzhou 310009, China; 3 Division of Biological Sciences, University of California, San Diego, 9500 Gilman Drive, La Jolla, CA 92093, USA

**Keywords:** p53 mutation, targeted therapy, gain-of-function, DNA-binding activity, MDM2/MDMX

## Abstract

**Simple Summary:**

Dysfunction of p53 has a significant impact on the resistance of tumor cells to various therapeutic agents. The major mechanisms causing p53 dysfunction include the MDM2/MDMX-mediated destabilization and inactivation of wtp53 and somatic p53 mutations. Despite an intensive search for therapeutic strategies to induce wtp53-like activities in tumor cells, their clinical efficacy and safety remain to be established. In this review, we summarize recent advances in the development of p53-targeting drugs, including their distinct mechanisms of action and potential in tumor therapy.

**Abstract:**

p53 plays a critical role in tumor suppression and is the most frequently mutated gene in human cancers. Most p53 mutants (mutp53) are missense mutations and are thus expressed in human cancers. In human cancers that retain wtp53, the wtp53 activities are downregulated through multiple mechanisms. For example, the overexpression of the negative regulators of p53, MDM2/MDMX, can also efficiently destabilize and inactivate wtp53. Therefore, both wtp53 and mutp53 have become promising and intensively explored therapeutic targets for cancer treatment. Current efforts include the development of small molecule compounds to disrupt the interaction between wtp53 and MDM2/MDMX in human cancers expressing wtp53 and to restore wtp53-like activity to p53 mutants in human cancers expressing mutp53. In addition, a synthetic lethality approach has been applied to identify signaling pathways affected by p53 dysfunction, which, when targeted, can lead to cell death. While an intensive search for p53-targeted cancer therapy has produced potential candidates with encouraging preclinical efficacy data, it remains challenging to develop such drugs with good efficacy and safety profiles. A more in-depth understanding of the mechanisms of action of these p53-targeting drugs will help to overcome these challenges.

## 1. Introduction

**Figure 1:**
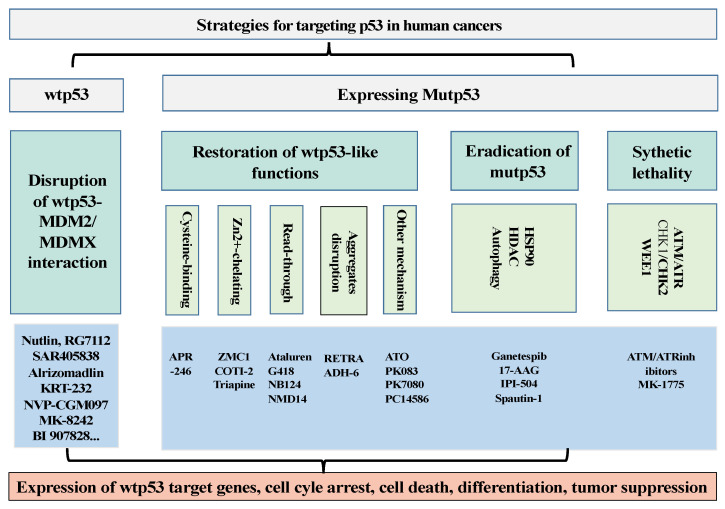
Summary of the strategies to target wtp53 and mutp53 in human cancers. For activating wtp53, drug development has been focused on strategies to disrupt the interaction between p53 and its negative regulators MDM2/MDMX. For human cancers expressing mutant p53, drug development has been focused on strategies to restore wild-type p53 activities to mutp53. In addition, drugs to eradicate mutp53 and induce synthetic lethality of tumor cells expressing p53 mutants have also been explored.

The tumor suppressor p53 plays a critical role in tumor suppression by directly regulating the expression of hundreds of genes important for the cell cycle, apoptosis, senescence, differentiation, and metabolism of normal cells [1]. Therefore, to evade the powerful tumor-suppressive activities of wtp53, the p53 gene has become the most frequently mutated gene in human cancers. The types of p53 mutations in human cancers include mostly missense mutations (70–80%), and less frequently, nonsense mutations (5–7%), frameshift mutations (5–7%), in-frame insertions/deletions (5%), and synonymous mutations (5%) [2]. p53 missense mutants often exhibit the loss of wild-type p53 functions (LOF), dominant-negative (DN) effects, and gain of functions (GOF). Many p53 missense mutants have lost wtp53-like DNA-binding activity while the remaining ones have retained residual activities. It remains unclear how partial LOF affects tumor cell survival in the therapeutic setting. p53 functions as a tetramer [3]. In the heterozygous state, mutp53 retains the ability to form tetramers with wtp53 and interferes with wtp53-dependent functions. The GOFs of p53 mutants promote tumorigenesis mainly through the interaction with other proteins involved in tumor development [4,5]. Considering the extensive and potent roles of p53 mutants in promoting tumorigenesis, p53 mutants have become an ideal therapeutic target for developing treatments for human malignancies (Figure 1).

## 2. p53 Targeting Therapies

### 2.1. Development of Drugs to Restore the Activities of wtp53 by Targeting p53–MDM2/MDMX Interaction

Two major negative regulators of wtp53 stability and activity, MDM2 and MDMX (MDM4), are overexpressed in human cancers [6]. MDM2 can negatively regulate the stability and activity of wtp53 through multiple mechanisms. MDM2, an E3 ubiquitin ligase for p53, is transcriptionally activated by p53 and provides a key negative regulatory loop to keep the levels of wtp53 low in normal cells without stresses [7]. In addition to inducing p53 degradation, the transcriptional activity of wtp53 is also inhibited by the interaction between the N-terminus of p53 and MDM2 [8,9,10]. Thirdly, MDM2 promotes the excretion of p53 from the nucleus and thus inhibits the activities of p53 as a transcriptional factor. Like MDM2, MDMX can also suppress the transcriptional activities of p53 by interacting with the N-terminus of p53 [11]. In addition, MDMX can stabilize MDM2, which has a very short half-life, and ultimately promotes p53 degradation [12,13,14]. Therefore, the disruption of the interaction between p53 and MDM2/MDMX in cancer cells overexpressing MDM2/MDMX could reactivate the wtp53-dependent tumor-suppressive activities in human cancer cells.

In the course of developing therapeutic strategies to disrupt the interaction between p53 and MDM2/MDMX, 4,5-dihydroimidazoline (Nutlin; Roche) was the first small molecule compound identified to specifically disrupt the p53–MDM2 interface and thus increase p53 stability and activity, leading to cell-cycle arrest, apoptosis, senescence, and differentiation of cancer cells [15,16]. However, because of its poor solubility, toxicity, and inadequate efficacy in clinical trials, the development of Nutlin as a p53 target drug was halted.

Other small molecule MDM2 inhibitors that have entered into clinical trials include RG7112, Idasanutlin (RG7388), SAR405838 (MI-77301), and Alrizomadlin (APG-115) (Table 1). RG7112 is a Nutlin analog with higher specificity for MDM2 and can induce more robust apoptosis of acute myeloid leukemia (AML) and chronic lymphocytic leukemia (CLL) cells than Nutlin 3a [17]. A Phase I study of RG7112 (NCT00623870) in patients with hematologic malignancies has shown that RG7112 does not affect p53 transcription but can inhibit p53 degradation, thereby activating p53 target genes and promoting cell-cycle arrest and apoptosis in leukemia cells. However, because RG7112 is an oral drug that requires a high dose, it can have serious toxic effects on gastrointestinal and bone marrow, especially on normal progenitors [17]. Among Nutlin derivatives, Idasanutlin is the most powerful so far. It suppresses the growth of SJSA1 human osteosarcoma harboring the wtp53 at a dose about four times lower than RG7112 [18]. Idasanutlin was found to be well tolerated and safe when used alone. However, the MIRROS (NCT02545283) study, a phase III clinical trial evaluating the efficacy of combinational therapy of idasanutlin + cytarabine in patients with relapsed or refractory (R/R) AML, showed disappointing results in its primary and secondary endpoints, overall survival (OS), and complete response (CR) [19]. Further phase I/II clinical trials are presently underway for the treatment of leukemia, glioblastoma, and other solid tumors in combination with immunotherapy, such as atezolizumab and chemotherapy (NCT04029688, NCT04589845, NCT03158389).

SAR405838, a highly specific spiro-oxindole MDM2 antagonist, induces robust activation of wtp53 in vitro and in vivo [20]. While two phase I clinical trials of SAR405838 (NCT01636479, NCT01985191) to treat malignancies have been completed, the findings of these clinical trials have yet to be published. Milademetan, an oral MDM2 inhibitor, has a binding pattern to MDM2 similar to SAR405838. In vivo studies have demonstrated that Milademetan activates p53 and inhibits tumor growth [21]. A first-in-human phase I study (NCT01877382) of Milademetan to treat solid tumors showed that the intermittent administration of the MDM2 inhibitor is beneficial for patients [22]. In addition, the MANTRA-2 trial (NCT05012397) assessed the safety and efficacy of Milademetan monotherapy in patients with metastatic solid tumors harboring wtp53 and MDM2 amplification [23]. The results indicated two unconfirmed partial responses (PRs) in 15 patients treated with Milademetan and a safety profile consistent with the previous phase I trial. The U.S. Food and Drug Administration (FDA) has designated Milademetan as an orphan drug for treating LFS.

Alrizomadlin is a novel oral MDM2-p53 inhibitor that achieves higher chemical stability than spiro-oxindole compounds by adding an ethyl group to the pyrrolidine. At the 2022 ASCO Annual Meeting, the latest data from the phase II clinical study of Alrizomadlin in combination with pembrolizumab to treat solid tumors were shown in a poster, showing the efficacy of the combinational therapy in patients with immuno-oncologic drug-resistant melanoma, including two cases (2/44) of CR, three cases (3/44) of partial response (PR), and a 13% ORR [24]. These data suggest that Alrizomadlin has the potential to treat patients with immunotherapy-resistant tumors.

Further structural optimization targeting a previously underutilized region of MDM2 (G58 “shelf”) led to the discovery of KRT-232, an oral MDM2 inhibitor with increased potency. KRT-232 is currently used in clinical trials to treat advanced malignancies, including AML, multiple myeloma, and glioblastoma. The efficacy of KRT-232 to treat wtp53 Merkel cell carcinoma (MCC) after the failure of anti-PD-1/L1 therapy was reported at the 2022 ASCO Annual Meeting, showing a confirmed ORR of 25%, a disease control rate (DCR) of 63%, and a median remission time of 4.1 months. Notably, one patient achieved CR confirmed by PET/CT after 2 years of treatment with sustained PR [25]. This study suggests that the upregulation of the p53 pathway would be a viable therapeutic strategy for MCC.

NVP-CGM097 is another structurally optimized MDM2 inhibitor. NCT01760525 is the first-in-human phase I study of NVP-CGM097 to treat solid tumors. The DCR was 39%, including one PR and 19 patients in SD with the tolerability of NVP-CGM097 manageable [26].

MK-8242, an oral MDM2 antagonist, showed tumor-suppressive activity in animal tumor models with wild-type p53 by disrupting the interaction between MDM2 and p53 [27]. Two Phase 1 clinical trials evaluating the clinical efficacy of MK-8242 as a monotherapy for advanced solid malignancies or in combination with cytarabine for AML have been completed (NCT01451437, NCT01463696). The results showed 1/24 PR, 1/24 CR, and 1/24 morphologic leukemia-free states [28]. Additionally, in the clinical trial enrolled with advanced solid tumors (NCT01463696), 27 patients with LPS had an mPFS of 237 days. MK-8242 also showed acceptable safety in this study [29].

At the 2022 ASCO Annual Meeting, Boehringer Ingelheim presented the results of a phase Ia/Ib, dose-escalation of MDM2 inhibitor BI 907828 to treat patients with solid tumors, including advanced/metastatic LPS. Among 41 enrolled patients with advanced LPS, a response of PR or SD was observed in 88.9% of patients with advanced DDLPS and 92.9% of patients with highly differentiated LPS (WDLPS). In the dose-escalation study, approximately 45% (5/11) of patients with DDLPS and approximately 50% (4/8) of patients with WDLPS had PFS ≥ 10.5 months [30]. In addition, this drug also showed a manageable safety profile, high plasma exposure, target engagement, and encouraging signs of antitumor activity in patients with advanced LPS.

Siremadlin, another MDM2 inhibitor, efficiently inhibited MDM2 and showed antitumor activity in cancer cell lines and wtp53 xenograft tumor models [31]. The drug is currently in 11 clinical trials for the treatment of advanced/metastatic soft-tissue sarcoma, AML, LPS, colorectal cancer, high-risk myelodysplastic syndrome (MDS), hepatic impairment, and other advanced solid and hematological wtp53 tumors. A phase I dose-escalation study (NCT02143635) in patients with TP53 wild-type advanced solid or hematologic cancers established controlled safety and preliminary activity, particularly in AML patients. Overall response rates (ORR) at the recommended dose for expansion (RDEs) were 10.3% in solid tumors and 4.2%, 20%, and 22.2% in AML with increasing concentrations [32]. A recent phase Ib dose-escalation study demonstrated that the combinational therapy with Siremadlin and Ribociclib (CDK4/6 inhibitor) exhibited manageable toxicity and antitumor activity in patients with radiologically progressive advanced WDLPS or DDLPS [33].

Overexpression of MDMX also occurs in several types of cancer, most notably melanoma [34]. To fully reactivate p53 in tumors with MDMX overexpression, dual targeting of MDM2/MDMX may be necessary. Staple peptides have an advantage over small molecule drugs in targeting protein–protein interactions (PPIs) due to enhanced binding affinity and stability for PPI interfaces. ALRN-6924 is the only dual MDM2 and MDMX inhibitor in clinical trials [35]. An open-label trial (NCT05622058) designed to evaluate the safety and efficacy of ALRN-6924 in breast cancer patients was prematurely terminated due to the findings that the first four patients treated had experienced grade 4 neutropenia and alopecia after cycle 1 and, as such, failed to meet the primary endpoint and the main secondary endpoint. VIP116 and its predecessor PM2 are novel stapled peptides targeting the MDM2/X–p53 interaction discovered by Lane’s group [36,37]. PM2 has been shown to avoid Nutlin-3 resistance and increase therapeutic effects by combining radiotherapy [38,39]. Furthermore, VIP116 with PEG-Stabilized Lipodisks promotes p53-mediated apoptosis with increasing peptide accumulation and decreasing toxicity [40]. Other dual MDM2 and MDMX inhibitors are being developed with significant anticancer activities in preclinical studies, including RO-2443 and optimized RO-5963 [41,42].

### 2.2. Development of Drugs to Target p53 Mutants (Mutp53)

When the p53 mutant protein is expressed, it plays multiple roles in driving tumorigenesis, including dominant-negative (DN) effects and gain of functions (GOF). Therefore, significant effort has been devoted to developing drugs to restore wtp53-dependent activities to mutp53 and inhibit the DN/GOF effects of mutp53. In principle, the drug that restores the wtp53 activities to mutp53 could simultaneously inhibit the DN/GOF effects of mutp53.

#### 2.2.1. Development of Drugs to Restore wtp53-Dependent Activities

Most p53 mutations can affect the transcriptional activities of wtp53, inducing the loss of DNA-binding activity directly or indirectly by the disruption of normal tetramerization of wtp53. Treatments with cysteine-binding compounds, Zn^2+^-chelating compounds, read-through drugs, and aggregation inhibitors are some approaches taken to restore wtp53 conformation, transcriptional activity, and tumor-suppressive activity.

##### Cysteine-Binding Compounds

Screening for compounds that suppress mutp53-dependent cell growth led to the discovery of PRIMA-1 [43]. The addition of a methyl group to PRIMA-1 led to APR-246, also known as PRIMA-1MET, which increased the pro-apoptotic activity and membrane permeability of the compound [44]. The proliferation of tumor cells harboring mutp53 is inhibited by the treatment of PRIMA1 and APR-246 both in vitro and in mouse xenograft tumor models [45,46,47]. In syngeneic mice, APR-246 can also suppress the growth of ascites tumors [48].

Mechanistically, methylene quinuclidinone (MQ), a Michael acceptor, is formed when the prodrug APR-246 is hydrolyzed in vivo. It can restore the mutp53 antitumor function by forming a covalent connection between the thiol groups of cysteines within the core DNA-binding domain. Experimental evidence suggests that Cys124 is a critical site for restoring the wtp53 activities to mutp53 [49]. MQ is also responsible for reactivating mutp53 by blocking its amyloid-like aggregation [50]. The off-target activities of these mutp53-targeting compounds also contribute to their antitumor effects. APR-246/MQ can form a covalent bond with the cysteine of glutathione, leading to increased levels of reactive oxygen species (ROS) and ferroptosis [51]. APR-246/MQ inhibits oxidoreductase enzyme thioredoxin reductase 1 (TrxR1), thioredoxin, and glutaredoxin, leading to oxidative stress [52,53]. APR-246 also appears capable of restoring the function of p53 homologs, including p63, TAP73a, TAp73b, and TAP63g, enhancing the capability of APR-246 to induce tumor cell death [54,55,56].

The efficacy and safety of APR-246 to treat malignant tumors were evaluated in a phase 1 clinical trial, the first-in-human trial for mutp53-targeting drugs (NCT00900614), showing the upregulation of p53 target genes with good safety, tolerance, and PK properties. In the phase II trial (NCT02098343), patients with recurrent platinum-sensitive high-grade ovarian cancer were treated with APR-246 in combination with carboplatin and pegylated doxorubicin at standard doses or carboplatin and pegylated doxorubicin. Using RECIST criteria, 3 of 21 evaluable patients had a confirmed CR, 10 had a confirmed PR, and 8 had SD [57]. APR-246 in combination with Azacitidine has produced encouraging results in patients with untreated TP53-mutated MDS and AML (NCT03072043). However, the data of the phase III trial (NCT03745716), which enrolled 154 patients with mutp53 MDS treated either with Azacitidine + APR-246 or Azacitidine alone, were disappointing with no significant difference between the two treatment groups [58].

##### Zn^2+^-Chelating Compounds

Zinc is essential for the correct folding of the central core domain of p53, and the absence of a zinc molecule in the central core can cause p53 to unfold and aggregate [59,60]. Immuno-precipitation experiments using the PAb1620 antibody showed that Zn^2+^ facilitated the refolding of mutp53 into the wild-type conformation [61]. By screening the NCI-60 cell line panel, Carpizo and his team found the chemical NSC319726 (ZMC1) that specifically reactivated the p53^R175H^ mutant likely by binding Zn^2+^ [62]. In addition to binding Zn^2+^, ZMC1 also binds Fe^2+^ and Cu^2+^, leading to increased ROS generation and oxidative stress [63,64].

COTI-2, a third-generation thiosemicarbazone identified by an in silico machine learning system, is active against a variety of malignant cell lines [65,66]. Unlike other thiosemicarbazones, COTI-2 appears capable of acting both dependently and independently of p53, resulting in cell death, restoration of p53 target gene expression, and activation of AMPK, while having no effect on intracellular Zn^2+^ levels [67]. COTI2 has been evaluated in a phase I trial (NCT02433626) for treating gynecological tumors with no evidence of tumor regression.

##### p53 Nonsense Mutation Read-Through Drugs

The p53 nonsense mutation accounts for about 10% of all p53 mutations. No tRNA can bind to the premature termination codon (PTC) in the p53 mRNA with nonsense mutation. Nonsense-mediated mRNA decay (NMD) efficiently identifies and degrades mRNAs containing PTC in open reading frames to prevent cell toxicity caused by the accumulation of truncated protein products [68]. Therefore, the strategy to restore the wtp53-like function is not feasible in this context. A more effective strategy is to induce the expression of the full-length p53 protein achieved by simultaneously targeting the induction of translational read-through and the inhibition of NMD.

In a cell-based screen, the read-through inducer Ataluren (PTC124) was discovered. This compound appears to be able to induce the expression of a functionally intact antimyotrophic protein. Insufficient evidence from relevant trials and concerns regarding its mode of action have led to its rejection by the FDA. Antibiotic aminoglycosides such as G418 (geneticin) and its derivative NB124 could induce read-through by elongating polypeptide chains by the insertion of a cognate tRNA. Synergistic effects were observed between NMD14, an NMD inhibitor, and G418 in inducing the expression of p53 target genes CDKN1A, BAX, and PUMA of human cancer cells harboring p53 nonsense mutations [69]. Unfortunately, aminoglycosides’ nephrotoxicity and ototoxicity severely restrict their potential therapeutic application [70].

##### Dispersion of p53 Aggregates

Mutp53 tends to form aggregates due to the exposure of adhesion sequences originally wrapped inside its hydrophobic core, and structural mutp53, such as R110P, R175H, R248Q, R249S, and R282W, can form amyloid aggregates [71]. In order to reactivate mutant p53, it is important to break up p53 aggregates and shift the folding equilibrium towards a “wild-type-like” state. The cell-penetrating peptide ReACp53 was utilized as an aggregation inhibitor of mutp53, particularly for R175H and R248Q mutants [72,73,74]. With its wild-type conformation and nuclear localization restored, p53 is able to promote apoptosis and cell-cycle arrest in cancer cells harboring p53 mutants in vitro and tumor suppression in vivo [73].

Taking advantage of an oligopyridylamide library known to suppress amyloid formation in Alzheimer’s disease, researchers found a tripyridylamide, denoted ADH-6, which can counteract the self-assembly of the aggregation-nucleating subdomain of mutp53 DBD. ADH-6 specifically recognizes and disrupts mutp53 aggregates in human cancer cells, reviving wtp53’s transcriptional activity and thus inducing cell-cycle arrest and apoptosis. Importantly, ADH-6 suppresses the growth of mutp53 xenografts without causing apparent toxicity to healthy tissues [75]. The clinical efficacy of these compounds is highly anticipated.

Members of the p53 family, such as p73 and p63, share a panel of target genes with wtp53. Compounds that activate these members of the p53 family could restore the Wtp53 activity because p73 and p63 are not frequently mutated in malignancies. However, mutant p53 can co-aggregate with p63 and p73, preventing their entry into the nucleus. Screening for compounds that can induce the expression of p53 target genes in mutp53-expressing cancer cells led to the discovery of the drug RETRA [76]. It activates the p73-dependent transcription and cell death by dismantling the mutp53-p73 aggregates. In a p73-dependent fashion, a small molecule compound, Prodigiosin, also promotes the expression of p53 target genes *p21*, *puma*, and *DR5* in mutp53 cells [77]. NSC59984 is a first-in-class small molecule drug that disrupts the mutp53-p73 complex and thus releases p73, inducing p73-dependent transcription in mutp53 cells. This compound also promotes the constitutive phosphorylation of ERK2 via ROS and MDM2-mediated ubiquitination and degradation of mutp53, suggesting a combinational therapy of this compound and ROS-inducing agents to treat mutp53 human cancers [78].

##### Other Compounds

Lu et al. demonstrated that arsenic trioxide (ATO) could restore structural mutp53 and identified 390 wtp53, the functions of which could be restored to various degrees by ATO [79]. ATO promotes the folding of structural mutp53 by functioning as an intramolecular glue that connects the LSH motif and β-sandwich motif [80]. In addition, single-cell sequencing of peripheral blood mononuclear cells (PBMCs) in a non-acute promyelocytic leukemia (APL) AML patient harboring a type 1 mutp53 after ATO monotherapy revealed upregulation of the classical p53 target gene and a significant reduction in minimal residual disease (MRD) in blood samples [79]. While this study supports the feasibility of restoring wtp53 function to mutp53 by ATO in human cancers, the low prevalence of type 1 mutp53 (0.1–0.2% in leukemias) as well as the toxicity and poor efficacy of ATO in solid tumors poses a challenge to larger-scale validation. Another compound, potassium antimony tartrate (PAT), was identified to restore 65 (1–2%) reversible temperature-sensitive (TS) mutp53 in preclinical experiments [81].

While the aforementioned compounds all have multiple mutp53 to target, more recent strategies have been to target either a single mutant or a subset of mutants with similar phenotypes. For example, the p53Y220C mutation is the eighth most prevalent p53 mutation across 12 different types of tumors [82,83]. The small molecule compounds PK083 and PK7080 were identified to target the Y220C mutation and restore wild-type conformation to mutp53, leading to tumor suppression [83,84]. PMV Pharmaceuticals used a similar strategy to develop its p53Y220C-targeting small molecule compound PC14586. Positive preliminary results of a phase I/II trial (NCT04585750) for the orally bioavailable PC14586 were presented at the last ASCO annual meeting. This study evaluated the safety, PK, PD, and preliminary efficacy of PC14586 in patients with advanced solid tumors with p53Y220C mutation. Among 21 patients with evaluable efficacy, 5 achieved PR; 30% of patients in the three highest dose groups achieved PR. In addition, the study found a reduction in the circulating tumor DNA and circulating tumor cells with p53Y220C mutation. In terms of safety, PC14586 was well tolerated. Therefore, PC14586 could represent a promising therapeutic agent to treat tumors with p53Y220C mutation [85]. Prof. Kevan M. Shokat’s team has recently discovered a new covalent inhibitor, KG13, that can restore the thermal stability of p53 y220c mutants to wild-type levels. H1299 (p53−/−) and U2OS (p53+/+) cells stably expressing p53 Y220C, as well as patient-derived NUGC-3 (p53Y220C/+) and BxPC-3 (p53−/−) cells, were treated with KG13 and analyzed for p53 target gene activation, cell growth inhibition, and increased caspase-3/7 activity [86].

Several other small molecule compounds have been identified to restore wtp53 activity in preclinical experiments. One such compound is RITA, a tiny chemical found to reactivate various mutp53, later also shown to interfere with the interaction between p53 and E6 [87,88,89,90]. In a p53 reporter screening assay, the C7-aryl piperlongumine derivatives KSS-9 and chetomin were found to bind to heat shock protein 40 (Hsp40), promoting its interaction with p53R175H to restore its wild-type conformation [91,92].

#### 2.2.2. To Develop Strategies to Eradicate Mutp53

Mounting evidence indicates that chemotherapy and radiotherapy of tumors can stabilize mutp53 and increase its GOF activity in promoting tumorigenesis and drug resistance. Therefore, eliminating mutp53 represents an intensively pursued strategy to inhibit GOF and drug resistance promoted by mutp53.

Hsp90 regulates the stability of many interacting proteins including mutp53 and its acetylation will weaken its interaction with target proteins [93]. HDAC is involved in the deacetylation of HSP90, and thus HDAC inhibitors can increase the acetylation levels of HSP90, leading to the destabilization of HSP90 target proteins such as mutp53 [94]. Ganetespib (STA-9090) is a novel resorcinolic triazolone compound targeting Hsp90 and binds to the N-terminal ATP-binding site of Hsp90. It can inactivate Hsp90 and induces cell-cycle arrest and apoptosis of cancer cells [95]. Disappointingly, ganetespib alone or in combination with other drugs (platinum, docetaxel, etc.) failed to improve the clinical outcome of patients. The geldanamycin derivative 17-AAG (Tanespimycin) and the 17-AAG hydroquinone hydrochloride salt derivative (IPI-504) have both shown antitumor activities and a well-tolerated profile in multiple clinical trials, including a phase III trial for multiple myeloma [96].

HSP40 proteins serve as co-chaperones for certain HSP70 proteins and modulate their functions by stimulating ATP hydrolysis in HSP70. Furthermore, HSP40 proteins are essential for protein translation, folding, unfolding, assembly, translocation, and degradation [97,98]. Statins were found to degrade conformational or misfolded mutp53 proteins via the mevalonate pathway-DNAJA1 (an HSP40 family member) axis in a Saos2 cell line expressing a p53R175H protein and in xenografts [99,100]. Trials NCT04767984 and NCT03560882 are currently investigating the antitumor activity of statins in patients stratified according to their p53 mutation status. These findings may lend support to the theory that the p53 status determines how effectively statins inhibit tumor growth. Statins also inhibit the HDAC6/Hsp90-dependent accumulation of mutp53 by inhibiting HDAC6 activity directly and disabling the mevalonate-RhoA axis [101,102]. In recent molecular docking studies, Tomoo Iwakuma’s group identified DNAJA1 inhibitors, PLTFBH and A11, which reduced DNAJA1 levels and then induced conformational mutp53 degradation, thereby inhibiting cancer cell migration [103,104].

Spautin-1, a small molecule compound that inhibits macroautophagy, can induce the degradation of a wide range of mutp53 proteins, including p53R175H/C/D, S241F, G245C, R248Q/W/L, E258K, R273H/L, R280K, and R282W [105]. Hence, targeting mutp53 for degradation by small molecule drugs is a promising strategy to improve the efficacy of therapy for tumors expressing mutp53.

### 2.3. Drugs Induce Synthetic Lethality with mutp53

Mutp53 frequently demonstrates oncogenic GOF activities by modulating many downstream signaling cascades in cancer cells. Hence, targeting critical mutp53 downstream pathways provides an alternate treatment for human cancers expressing mutp53. Cancer cells may develop a secondary survival reliance due to an oncogenic mutation or tumor suppressor deficiency, when the secondary survival pathway is also mutated, leading to a condition known as synthetic lethality [106]. For example, the inactivation of the ATR/CHK1, ATM/CHK2, or p38MAPK/MK2 pathway has been shown to confer synthetic lethality with p53-deficiency of cancer cells [107].

Preclinical studies of the WEE1 inhibitor MK-1775 (AZD1775) have shown efficacy against head and neck tumors expressing mutp53, either alone or in combination with cisplatin [108]. Currently, MK1775 has been tested in multiple clinical trials to examine its efficacy to treat tumors with p53 deletions or mutations. In the NCT01357161 trial, MK-1775 enhanced the effectiveness of carboplatin/paclitaxel chemotherapy in women with platinum-sensitive ovarian cancer expressing mutp53, with a modest clinical benefit as indicated by PFS (median 34.14 vs. 31.86 weeks) [109]. In the NCT01164995 trial, MK-1775 enhanced the efficacy of carboplatin to treat resistant or refractory ovarian cancer expressing mutp53 [110]. In the NCT02272790 trial, the efficacy and safety of MK-1775 in combination with chemotherapy (gemcitabine, paclitaxel, carboplatin, or pegylated liposomal doxorubicin (PLD)) for platinum-resistant ovarian cancer were examined, showing promising results as reported in the 2019 ASCO Annual Meeting. However, this combinational therapy caused very notable hematologic toxicity among the whole cohort [111]. Therefore, the regimens of MK-1775 need to be optimized to improve the safety of the treatment.

### 2.4. Cancer Immunotherapy for Mutp53

Adoptive cell transfer therapy (ACT) refers to the isolation of immunologically active cells from tumor patients, their expansion and functional characterization in vitro, and then their infusion back to the patient for the purpose of killing the tumor directly or stimulating the body’s immune response to kill the tumor cells.

The vast variety of P53 missense mutations in human malignancies generates a large number of potential neoantigens that may stimulate mutant p53-specific immune responses. Using novel high-throughput techniques, a recent study has evaluated the response of T cells within tumors to p53 mutations, showing that hotspot mutations can be processed and presented by HLA on the surface of tumor cells. p53 hotspot mutations have a complex immunogenicity, which is influenced by many factors such as the stability of mutp53, HLA alleles expressed on tumor cells or APC, and TCR affinity. In the future, it is anticipated that more in-depth studies can be conducted on larger patient cohorts [112].

## 3. Conclusions

p53 is a crucial tumor suppressor and is mutated in about 50% of human cancers, making both wtp53 and mutp53 appealing therapeutic targets for cancer treatment. The strategies to induce wtp53-like activities in human cancer cells are complex and must take into consideration different scenarios such as the loss of function of wtp53, and the dominant-negative and gain of oncogenic functions of mutp53. Apparently, more precision in developing drugs targeting individual mutp53 or mutp53s of similar features is required. Strategies to reverse the DN and GOF effects associated with some types of mutp53 have been shown to boost therapeutic responses and inhibit tumor growth in preclinical models. However, despite extensive clinical testing of these compounds, the efficacy and safety of these drugs remain challenging for their clinical application. Considering the potent roles of p53 in both tumor suppression and aging, in the course of developing p53-targeting drugs, one important balance to maintain is to activate wtp53-dependent tumor suppression without inducing p53-dependent aging. A more in-depth understanding of the mechanism of action of these drugs on various p53-dependent functions is important to improve their efficacy and safety.

## Figures and Tables

**Table 1:** Summary of clinical trials of drugs to activate wild-type activities of p53 in human cancers.

Drug Name	Combination with Other Therapy	Type of Malignancies	Clinical Trials	Clinical Trial ID
RG7112		Hematologic	1	NCT00623870
Idasanutlin	Cytarabine	AML	3	NCT02545283
	Chemotherapy/ Venetoclax	AML Solid tumors	2	NCT04029688
		Solid tumors	2	NCT04589845
		Glioblastoma	2	NCT03158389
SAR405838		Malignant neoplasm	1	NCT01636479
		Malignant neoplasm	1	NCT01985191
Milademetan		Advanced solid tumor, Lymphoma	1	NCT01877382
		Solid tumor	2	NCT05012397
Alrizomadlin	Pembrolizumab	Metastatic melanomas Advanced solid tumors	2	NCT03611868
KRT-232		MCC	2	NCT03787602
NVP-CGM097		Solid tumors	1	NCT01760525
MK-8242	Cytarabine	AML	1	NCT01451437
		Solid tumors	1	NCT01463696
BI 907828		Solid tumors	1	NCT03449381
Siremadlin		Advanced solid tumors Hematological neoplasm	1	NCT02143635
	LEE011	Solid tumors	1	NCT02343172
ALRN-6924		Advanced solid tumors	1	NCT05622058
APR-246		Refractory hematologic tumor Prostate cancer	1	NCT00900614
	Carboplatin+ Pegylated Liposomal Doxorubicin Hydrochloride (PLD)	Platinum sensitive recurrent high-grade serous ovarian Cancer with p53 Mutations	2	NCT02098343
		Myeloid Neoplasms with p53 mutations	2	NCT03072043
	Azacitidine	MDS with p53 mutations	3	NCT03745716
COTI-2	Cisplatin	Advanced solid tumors with p53 mutations	1	NCT02433626
Tanespimycin	Bortezomib	Multiple myeloma with p53 mutations	3	NCT00514371
Atorvastatin Calcium		Colorectal Carcinoma Ulcerative Colitis with p53 mutations	2	NCT04767984
Atorvastatin		Advanced solid tumors with p53 mutations	1	NCT03560882
MK-1775	Paclitaxel+ Carboplatin	Platinum-sensitive Ovarian Tumors with p53 Mutations	2	NCT01357161
	Carboplatin	Epithelial ovarian cancer with p53 Mutation	2	NCT01164995
	Paclitaxel/Carboplatin/Gemcitabine/PLD	Ovarian, Fallopian Tube, Peritoneal cancer with p53 mutations	2	NCT02272790

AML: Acute Myeloid Leukemia; MDS, myelodysplastic syndrome.
